# Supplementary material for: Estrogen receptor α drives pro-resilient transcription in mouse models of depression
Source: Nat Commun. 2018 Mar 16;9:1116. doi: 10.1038/s41467-018-03567-4 (PMC5856766; doi:10.1038/s41467-018-03567-4)
Supplement: Supplementary file 1 — Supplementary Information(PDF 10695 kb) [file 41467_2018_3567_MOESM1_ESM.pdf]

Supplemental Figure 1

Lorsch *et al.*

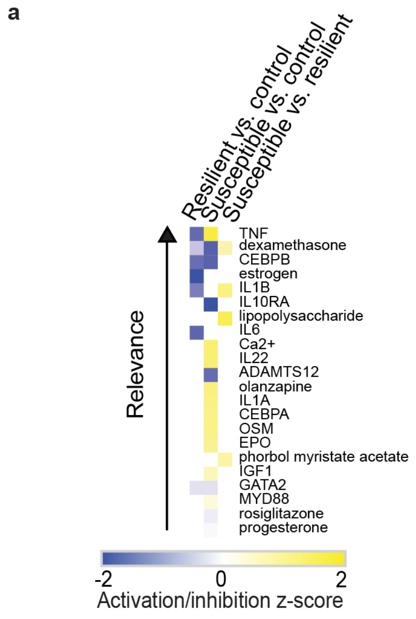

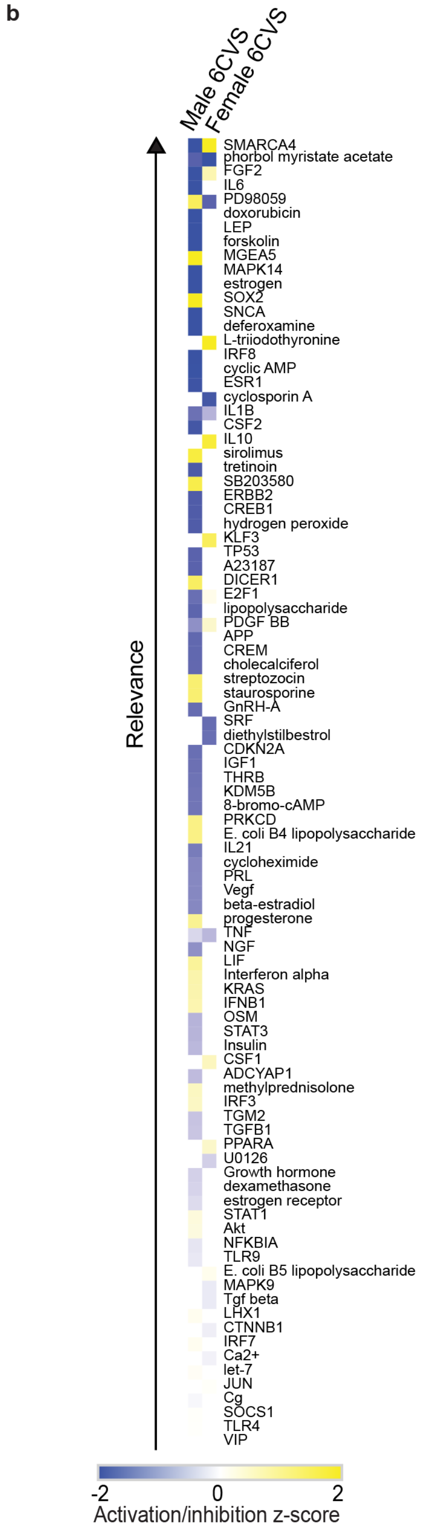

Supplementary Figure 1: Predicted upstream regulators of transcriptional changes following CSDS in PFC and CVS in NAc. (**a**) Findings from IPA upstream regulator analysis of differentially expressed genes (log fold change > 1; $P < 0.05$) in PFC following CSDS reveals that *ESR1* is not a predicted upstream regulator of differentially expressed genes in this brain region. (**b**) IPA upstream regulator analysis of differentially expressed (log fold change > 1; $P < 0.05$) genes in NAc following 6 day of CVS. *ESR1* is predicted to be significantly inhibited (z-score = -1.80; $P < 1.76 \times 10^{-2}$) following CVS in males with no predicted regulation for female CVS.

Supplemental Figure 2 Lorsch *et al.*

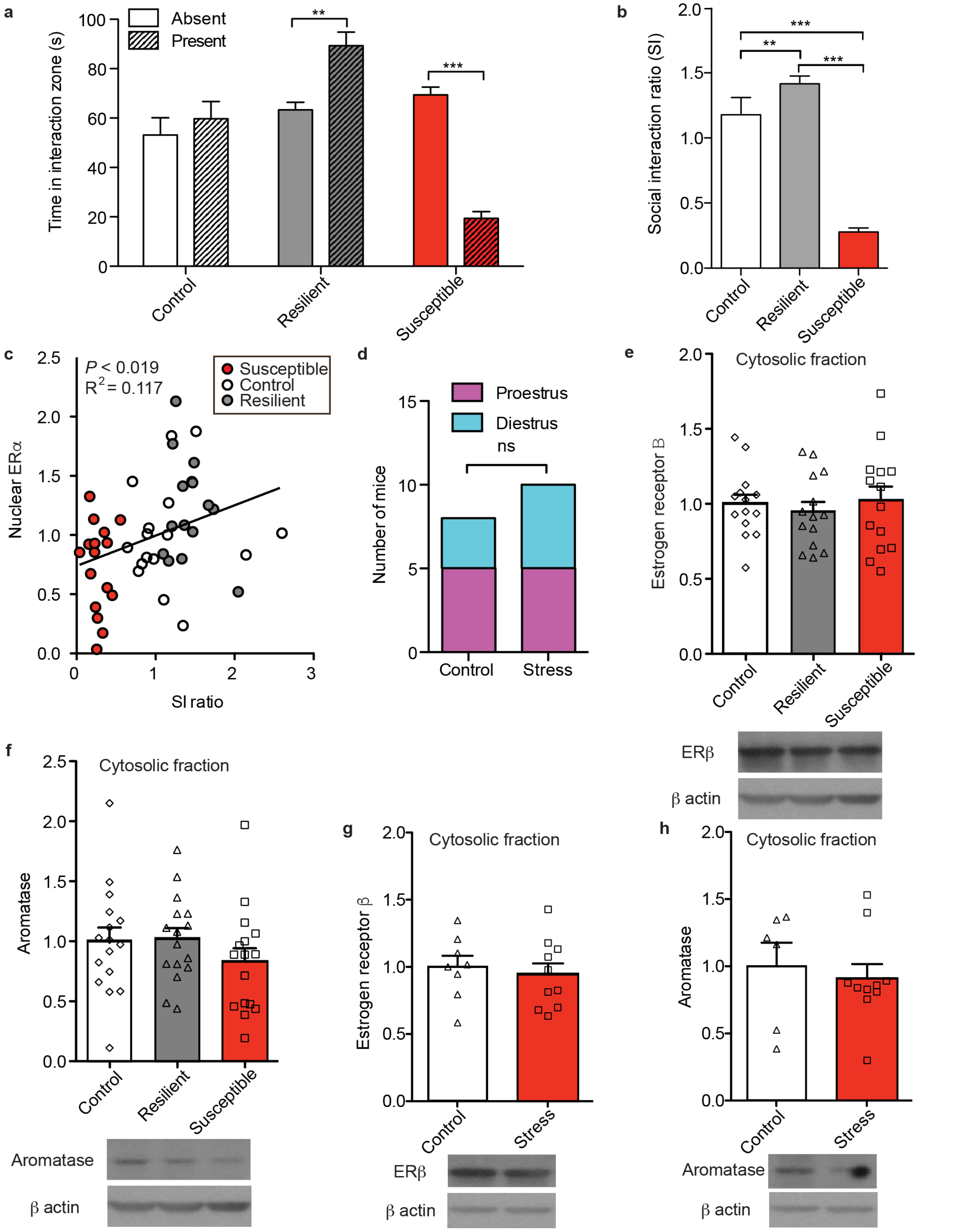

Supplementary Figure 2: Characterization of stress phenotypes and their effect on estrogen-related protein expression in NAc. In male mice used for protein analysis (**a**) there are significant group differences in time spent in the interaction zone in the social interaction test ($\chi^2(5) = 44.96$, $P < 0.001$, n= 16). Control, susceptible, and resilient mice show different preferences for the interaction zone when the CD1 aggressor is present ($\chi^2(2) = 28.53$, $P < 0.001$), but not when the CD1 aggressor is absent ($\chi^2(2) = 3.41$, $P < 0.182$). Resilient mice spend more time in the interaction zone when the CD1 mouse is present ($P < 0.002$) while susceptible mice spend more time in the interaction zone when the CD1 mouse is absent ($P < 0.001$). (**b**) There is an effect ($\chi^2(2) = 34.47$, $P < 0.001$, n= 16) of CSDS phenotype on SI with susceptible mice showing lower SI ratios than both resilient ($P < 0.001$) and control ($P < 0.001$) phenotypes, and resilient mice showing higher SI than controls ($P < 0.008$). (**c**) Across all phenotypes, larger SI is positively associated with higher nuclear ERα protein levels in NAc as determined by linear regression analysis ($F_{(1,45)} = 5.94$, $P < 0.019$; R^2=0.117, n=47). (**d**) Estrus cycle analysis reveals similar distributions of proestrus and diestrus mice in CVS females utilized for protein analysis ($X^2(1,, N=18) = 0.281$, $P < 0.596$). CSDS phenotype has no effect on the expression of (**e**) ERβ ($F_{(2,39)} = 0.279$, $P < 0.758$; n = 14) or (**f**) aromatase ($F_{(2,45)} = 0.961$, $P < 0.390$; n = 16) expression in the cytosolic fraction of male mice. CVS has no effect on the expression of (**g**) ERβ ($t_{(16)} = 0.48$, $P < 0.641$; n = 8,10) or (**h**) aromatase ($t_{(14)} = 0.47$, $P < 0.647$; n = 6,10) in the cytosolic fraction of female mice. Error bars ± SEM. **a, b** Kruskall-Wallis test. **d** Chi-Square test. **e, f** One-way ANOVA. **g, h** two tailed t-test. $^{**}P < 0.01$, $^{***}P < 0.001$, post-hoc independent samples Mann-Whitney. ns = not significant.

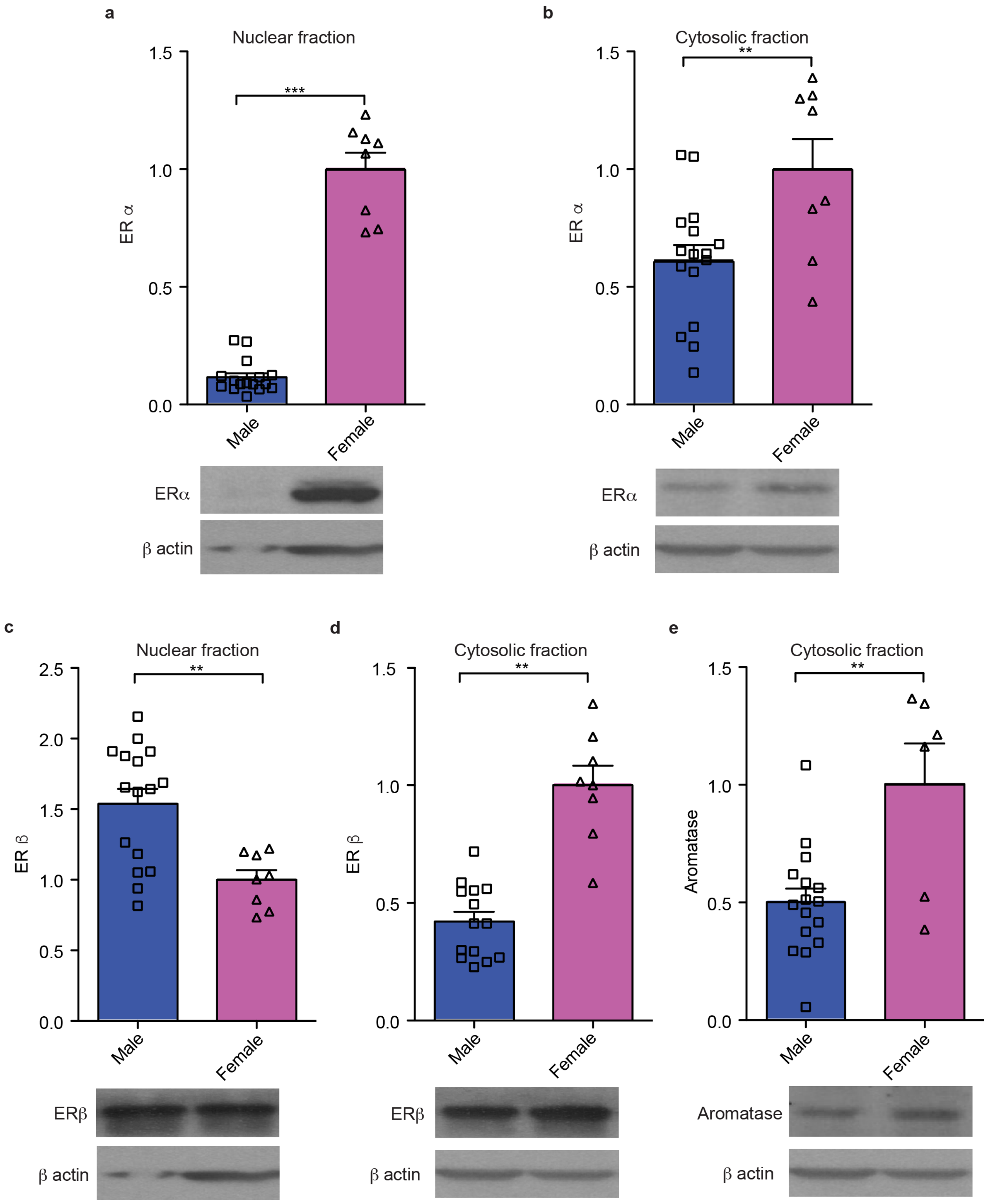
a
Nuclear fraction
***
1.5
1.0
0.5
0.0
ER α
Male
Female
ERα
β actin
b
Cytosolic fraction
**
1.5
1.0
0.5
0.0
ER α
Male
Female
ERα
β actin
c
Nuclear fraction
**
2.5
2.0
1.5
1.0
0.5
0.0
ER β
Male
Female
ERβ
β actin
d
Cytosolic fraction
**
1.5
1.0
0.5
0.0
ER β
Male
Female
ERβ
β actin
e
Cytosolic fraction
**
1.5
1.0
0.5
0.0
Aromatase
Male
Female
Aromatase
β actin

Supplementary Figure 3: Baseline sex differences in the expression of estrogen receptors and aromatase in the mouse NAc. Male mice exhibit lower levels ERα (**a**) in the nucleus ($U = 0$, $P < 0.001$, n = 16,8) and (**b**) cytosol ($t_{(22)} = 3.012$, $P < 0.006$; n = 16,8) when compared to female mice at baseline. (**c**) Male mice show more ERβ in the nucleus than female mice ($U = 19$, $P < 0.006$, n = 16,8). In control mice in the cytosolic fraction, both (**d**) ERβ ($t_{(20)} = 6.93$, $P < 0.001$; n = 14,8) and (**e**) aromatase ($t_{(20)} = 3.54$, $P < 0.002$; n = 16,6) are expressed at higher levels in female than male mice. **a, c** independent samples Mann-Whitney. **b, d, e** two tailed t-test. $^{**}P < 0.01$, $^{***}P < 0.001$.

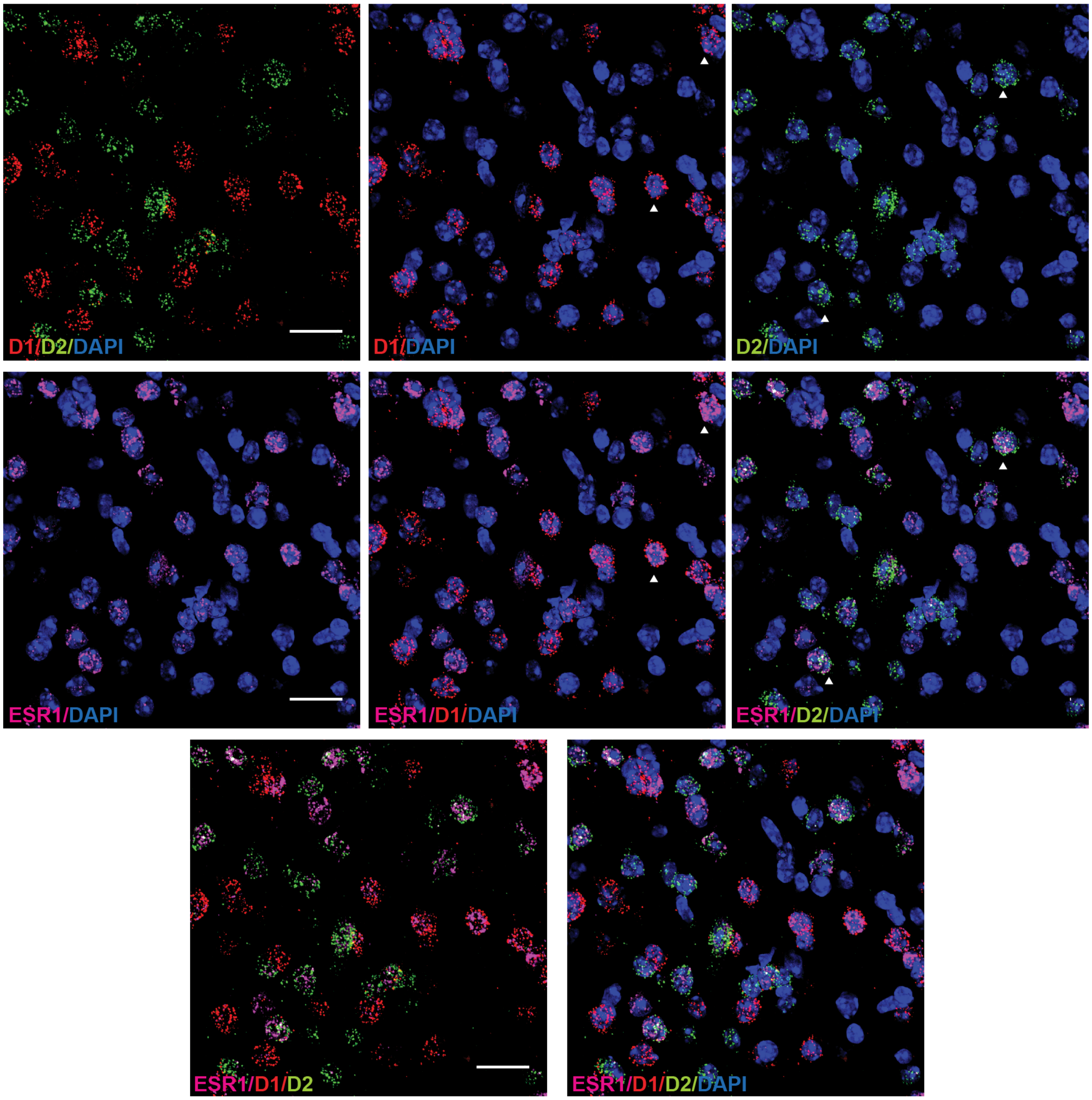

Supplementary Figure 4: Localization of virus-dependent *ESR1* mRNA within D1 and D2 expressing neurons in NAc. RNAscope probes for human *ESR1* (magenta), which were expressed as a result of the AAV-ESR1 injection, overlap with probes for both D1 (red) and D2 (green) positive neurons. Scale bars = 20 μM.

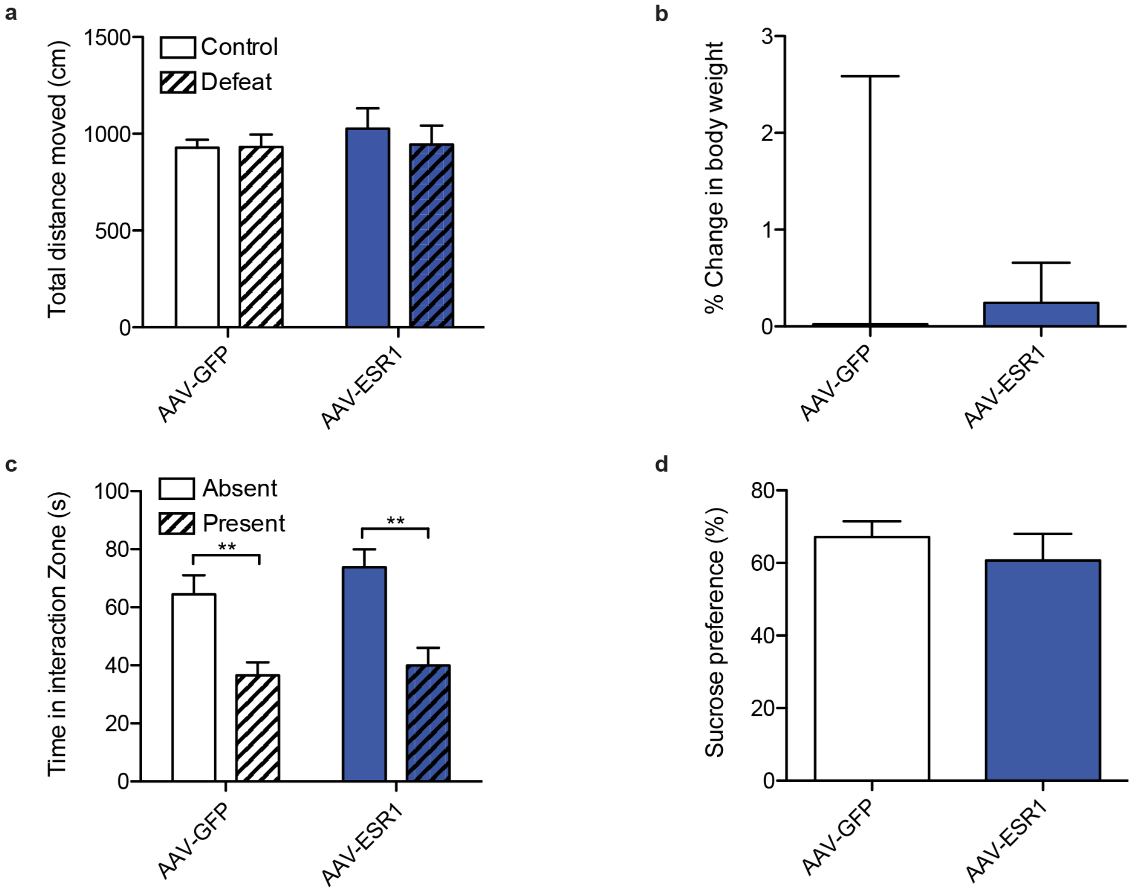

Suplementary Figure 5: Effects of ERα overexpression in NAc and PFC in male mice. (**a**) There is no effect of virus ($F_{(1,31)} = 0.499$, $P < 0.485$; n = 10,9,8,9) or defeat ($F_{(1,31)} = 0.238$, $P < 0.629$) on locomotion and (**b**) no effect of virus on change in body weight 3.5 weeks after viral infusion ($U = 9$, $P < 0.548$, n = 5). In mice undergoing CSDS following viral infusion in PFC (**c**) whether a CD1 aggressor mouse is present ($F_{(1,18)} = 27.88$, $P < 0.001$, n = 10), but not virus ($F_{(1,18)} = 1.15$, $P < 0.280$), has a significant effect on time spent in the interaction zone in the social interaction test and (**d**) ERα overexpression has no effect on sucrose preference ($t_{(19)} = 0.774$, $P < 0.445$; n = 11,10). **a,** Two-way ANOVA**. b,** independent samples Mann-Whitney. **c,** Mixed-Models ANOVA. **d,** two tailed t-test. ** $P < 0.01$ post-hoc independent samples Mann-Whitney.

a

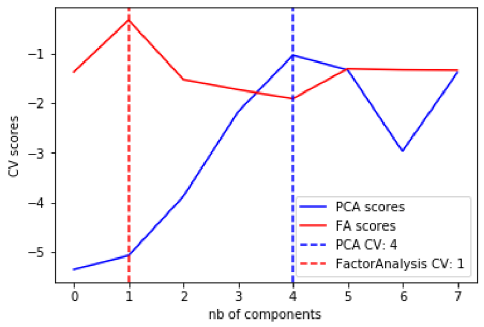

b

Behavioral Measure	Factor 1
Latency to Immobility	-0.111685
Overall Sucrose	-0.072095
Sucrose 1	-0.200006
Sucrose 2	-0.031671
Sucrose 3	-0.133491
Sucrose 4	-0.069307
NSF Home	-0.249502
NSF Novel	0.188730

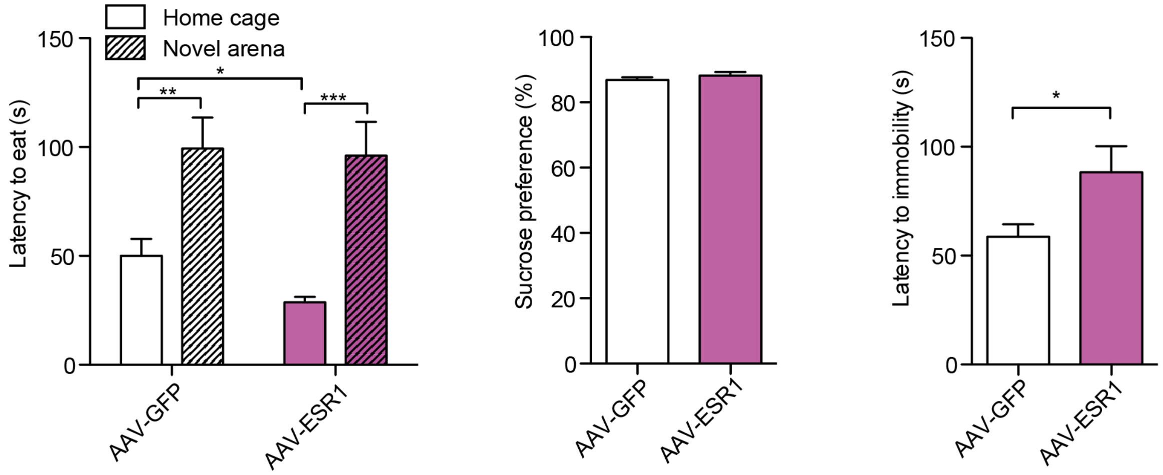

Suplementary Figure 6: Female factor analysis and behavioral differences in unstressed controls. (**a**) Comparison of communalities (observed variance) for different numbers of components in exploratory factor analysis and principal components analysis reveals maximum likelihood for a single factor in exploratory factor analysis. (**b**) Variable loading for factor 1. (**c**) In unstressed female mice there is a significant difference between groups in latency to eat in the novelty suppressed feeding test ($\chi^2(3) = 22.31$, $P < 0.001$, n= 11,12). Both AAV-GFP ($P < 0.009$) and AAV-ESR1 ($P < 0.001$) injected mice take longer to feed in the novel arena but virus only affected latency to eat in the home cage ($P < 0.016$). While there is no significant difference for unstressed females in (**d**) sucrose preference ($t_{(21)} = 0.932$, $P < 0.362$; n = 11,12), (**e**) AAV-ESR1 injected mice show a higher latency to immobility in the forced swim test ($U = 26.5$ $P < 0.027$, n = 11,12). **c,** Kruskal-Wallis test**. d,** two tailed t-test**. e,** independent samples Mann-Whitney. $^{*}P < 0.05$, $^{**}P < 0.01$, $^{***}P < 0.001$ in **c** post-hoc independent samples Mann-Whitney.

Supplemental Figure 7 Lorsch *et al.*

a

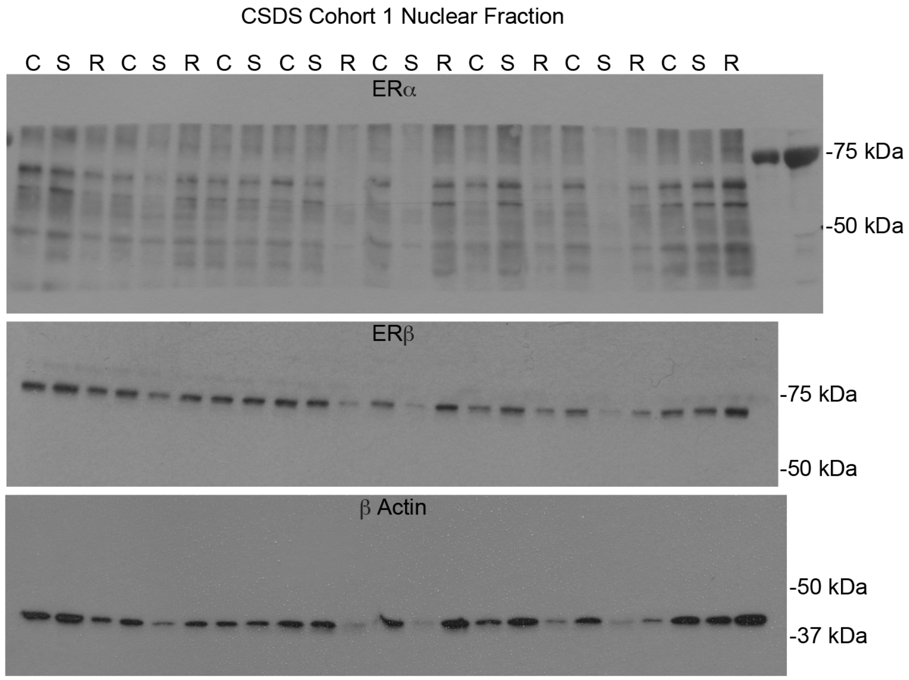

b

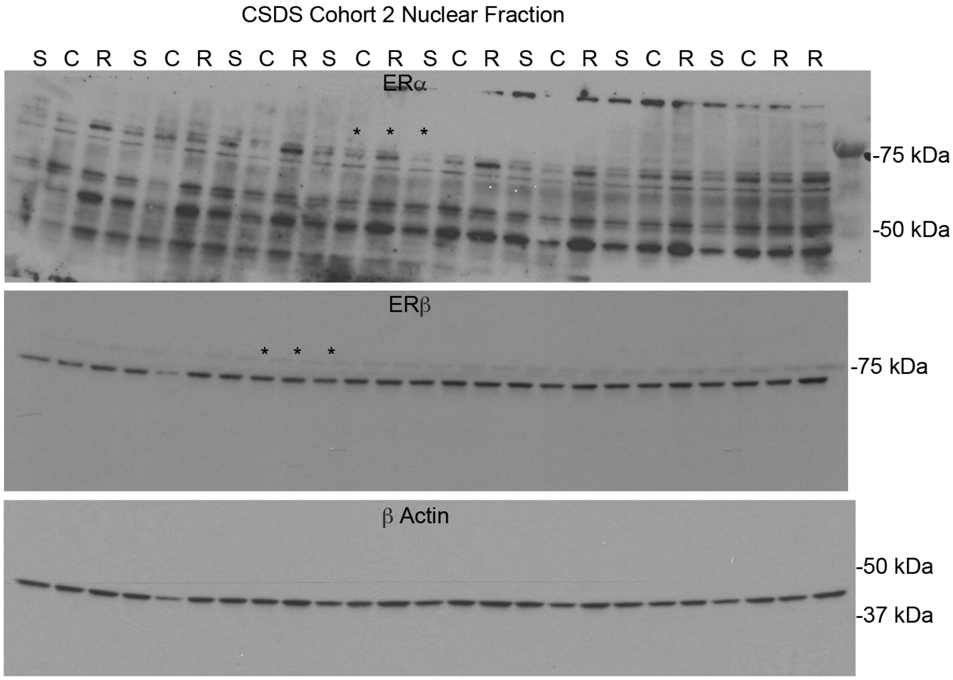

Supplementary Figure 7: Full western blots for male CSDS nuclear fraction. Stars identify lanes that were cropped to be featured in main figures.

Supplemental Figure 8 Lorsch *et al.*

a

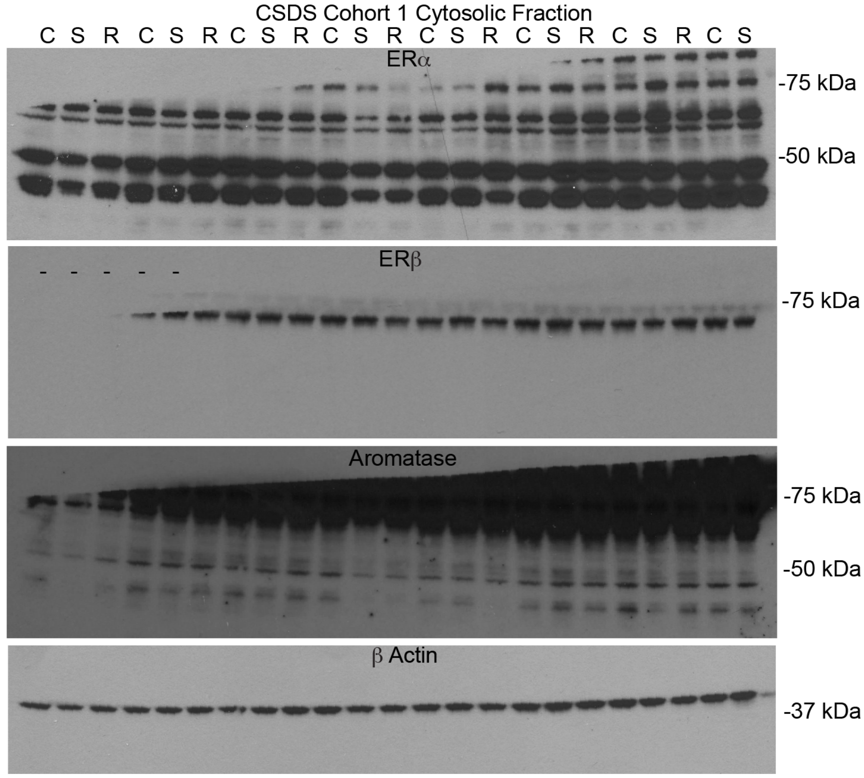

b

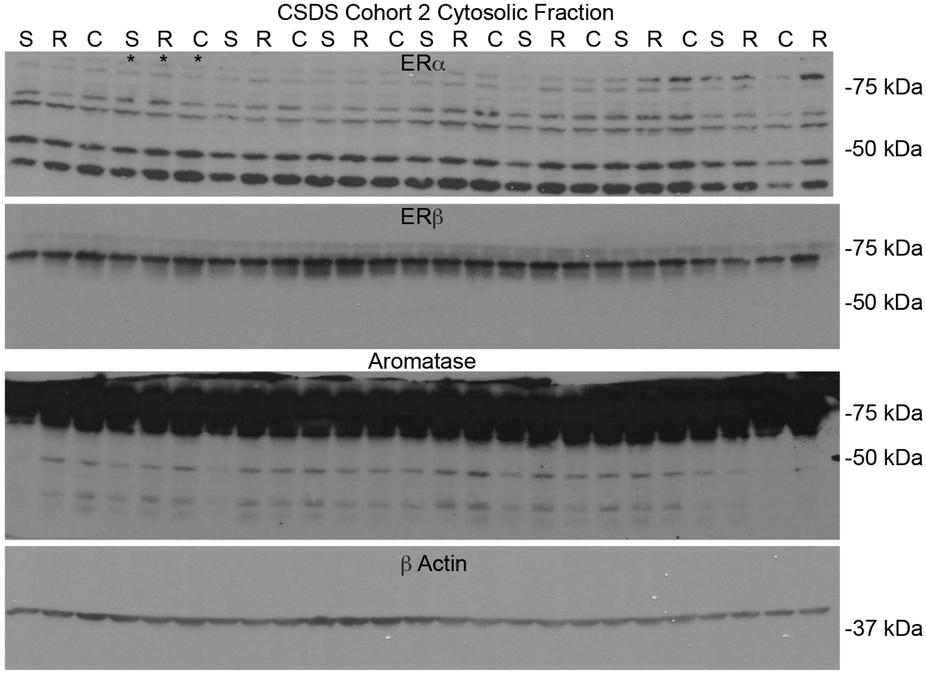

Supplementary Figure 8: Full western blots for male CSDS cytosolic fraction. Stars identify lanes that were cropped to be featured in main figures. Minus signs indicate that lanes were not utilized in analysis.

Supplemental Figure 9 Lorsch *et al.*

a

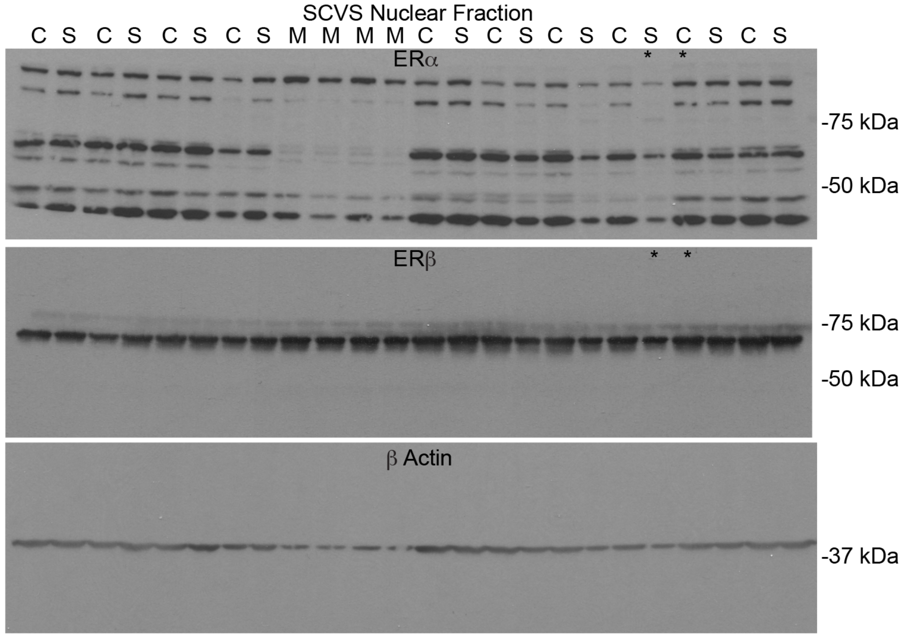

b

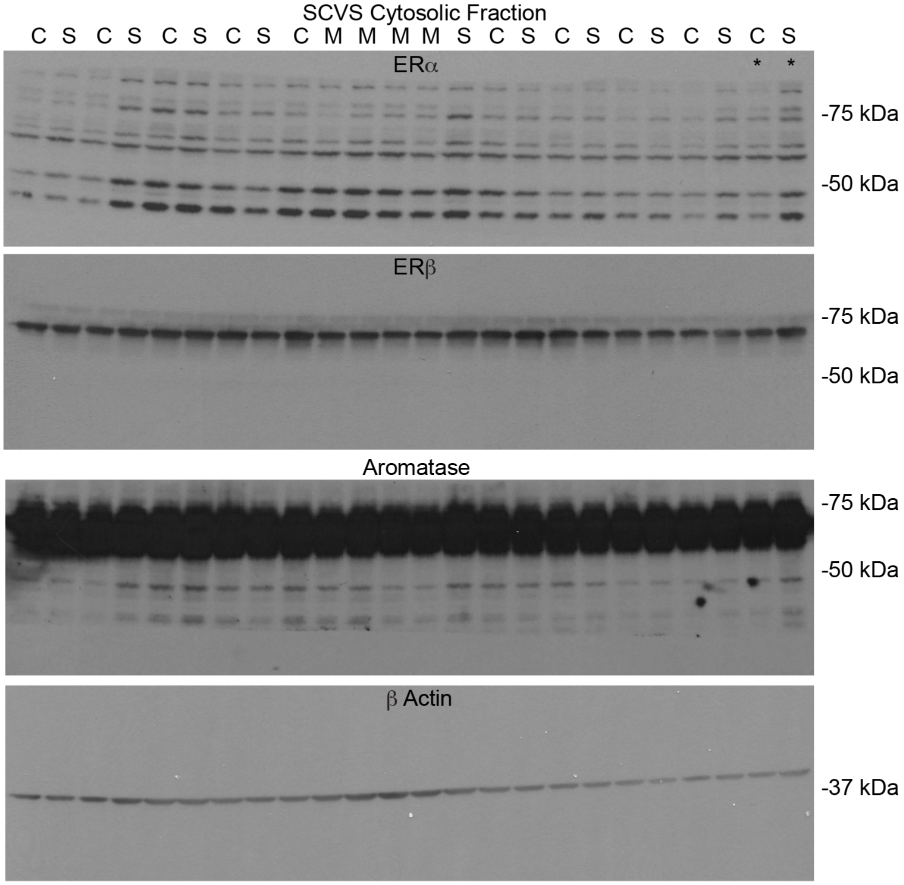

Supplementary Figure 9: Full western blots for female CVS. Stars identify lanes that were cropped to be featured in main figures.
